# An important call: Suggestion of using IL-10 as therapeutic agent for COVID-19 with ARDS and other complications

**DOI:** 10.1080/21505594.2023.2190650

**Published:** 2023-03-16

**Authors:** Li-Jane Shih, Chun-Chun Yang, Min-Tser Liao, Kuo-Cheng Lu, Wan-Chung Hu, Chih-Pei Lin

**Affiliations:** a Department of Medical Laboratory, Taoyuan Armed Forces General Hospital, Taoyuan City, Taiwan; b Graduate Institute of Medical Science, National Defense Medical Center, Taipei City, Taiwan; c Department of Laboratory Medicine, Taipei Tzu Chi Hospital, Buddhist Tzu Chi Medical Foundation, New Taipei City, Taiwan; d Department of Pediatrics, Taoyuan Armed Forces General Hospital, Taoyuan, Taiwan; e National Defense Medical Center, Department of Pediatrics, Tri-Service General Hospital, Taipei, Taiwan; f Division of Nephrology, Department of Medicine, Fu-Jen Catholic University Hospital, New Taipei City, Taiwan; g Department of Clinical Pathology, Taipei Tzu Chi Hospital, Buddhist Tzu Chi Medical Foundation, New Taipei City, Taiwan; h Department of Biotechnology, Ming Chuan University, Taoyuan, Taiwan

**Keywords:** Tr1, IL-10, virus, COVID-19, ARDS

## Abstract

The global coronavirus disease 2019 (COVID-19) pandemic has a detrimental impact on public health. COVID-19 usually manifests as pneumonia, which can progress into acute respiratory distress syndrome (ARDS) related to uncontrolled TH17 immune reaction. Currently, there is no effective therapeutic agent to manage COVID-19 with complications. The currently available anti-viral drug remdesivir has an effectiveness of 30% in SARS-CoV-2–induced severe complications. Thus, there is a need to identify effective agents to treat COVID-19 and the associated acute lung injury and other complications. The host immunological pathway against this virus typically involves the THαβ immune response. THαβ immunity is triggered by type 1 interferon and interleukin-27 (IL-27), and the main effector cells of the THαβ immune response are IL10-CD4 T cells, CD8 T cells, NK cells, and IgG1-producing B cells. In particular, IL-10 exerts a potent immunomodulatory or anti-inflammatory effect and is an anti-fibrotic agent for pulmonary fibrosis. Concurrently, IL-10 can ameliorate acute lung injury or ARDS, especially those caused by viruses. Owing to its anti-viral activity and anti-pro-inflammatory effects, in this review, IL-10 is suggested as a possible treatment agent for COVID-19.

## Introduction

Recently, the coronavirus Severe Acute Respiratory Syndrome Coronavirus 2 (SARS-CoV-2) has caused a severe global outbreak of coronavirus disease 2019 (COVID-19). COVID-19 is a severe respiratory-related disease that usually causes pneumonia. WHO designated the disease as COVID-19. The structure of SARS-CoV-2 is very similar with that of the severe acute respiratory syndrome (SARS) virus; both of them bind to the receptor of angiotensin-converting enzyme 2 (ACE2) to infect pulmonary epithelial cells. SARS-CoV-2 can cause severe complications, including ARDS, with a very high mortality, especially in the elderly population. As of 14 December 2022 650 million COVID-19 cases and more than 6.6 million associated deaths have been reported globally [1] [2].

The clinical manifestations of COVID-19 include cough, fever, dyspnoea, and respiratory pain. Upper respiratory symptoms and diarrhoea are also noted but are less common. The SARS-CoV-2 infection can progress into severe respiratory complications, such as pneumonia or acute lung injury [3]. Progression to acute lung injury or acute respiratory distress syndrome (ARDS) is associated with a high mortality. Comorbidities related to higher mortality include cardiovascular disease, diabetes mellitus, hypertension, obesity, chronic lung disease, chronic liver disease, chronic kidney disease, smoking, and malignancy. Several laboratory findings have been related to worse outcomes, including lymphopenia, increased prothrombin time, and elevated levels of liver enzymes, lactate dehydrogenase, inflammatory markers (e.g. C-reactive protein [CRP] and ferritin), D-dimer, troponin, creatine phosphokinase, and creatinine with acute kidney injury. Some severe COVID-19 patients have laboratory findings of an overt inflammatory response, cytokine release syndrome, continuous fevers, elevated inflammatory markers (e.g. CRP and ferritin), and upregulated levels of pro-inflammatory cytokines (e.g. TNF alpha, IL-1, and IL-6). Thus, the use of IL-6 inhibitors to treat COVID-19 has been attempted, with a success rate of approximately 32% [4]. Chest CT of COVID-19 patients can show a peripheral distribution, ground-glass appearances, fine reticular patterns, vascular thickening, and reverse halo mark. Laboratory diagnostic requires a positive RT-PCR nucleic acid test results.

However, to date, there is no effective therapeutic agent to treat this detrimental infectious disease. An anti-viral agent, remdesivir, has been reported to have a 30% response rate for treating COVID-19. Nevertheless, better treatment strategies for SARS-CoV-2 infection and ARDS are required [5].

## Overview of ARDS

ARDS is a syndrome that is related to an overactive innate immune response. In ARDS, hyperactive macrophages and neutrophils release pro-inflammatory cytokines, causing cytokine storm and severe immune cell infiltration in the lung. In the later stage, regulatory cells (Treg) become dominant and mediate pulmonary fibrosis to cause a sequel to ARDS. Because ARDS is a consequence of overactive TH17-like innate immunity, its management should focus on inhibiting the overactive host innate immune response. Immune modulation by suppressing the production of pro-inflammatory cytokines could be a promising strategy. However, there is no successful treatment strategy for ARDS; thus, its prognosis remains very poor. Infectious agents, such as SARS-CoV, can cause ARDS [6–8]. In addition, SARS-CoV can downregulate type 1 interferon signalling, which suppresses the anti-viral adaptive immunity [9,10]. This downregulation is important to the pathophysiology of COVID-19.

ARDS is often a complication of sepsis caused by bacterial infection. However, virus-induced ARDS is also seen. The SARS-CoV or influenza virus can also cause ARDS as a complication of these lethal viral infections [11,12]. ARDS is a major complication of severe COVID-19 disease and can manifest shortly after the onset of dyspnoea. If SARS-CoV2 infection induces ARDS, the mortality rate can become approximate 40%. In a study of 201 hospitalized patients with COVID-19 in Wuhan, 41% developed ARDS, and age >65 years, diabetes mellitus, and hypertension were associated with increased risk of ARDS [13]. In another study of 2741 patients who were hospitalized for COVID-19 in New York City, 24% (*n* = 665) died or were discharged to hospice care, including 241 patients who were not treated in an intensive care unit [14]. Moreover, among the 749 patients who received intensive care (27% of the total cohort), 647 received mechanical ventilation; of these patients, 60% died, 13% were still ventilated, and 16% were discharged. In a study of 138 patients, ARDS developed in 20% of the patients at an average of eight days after the onset of symptoms; mechanical ventilation was required in 12.3% of the patient [15]. In large studies conducted in the United States, 12–24% of hospitalized patients required mechanical ventilation [14,16].

## Host immunological pathways against viral or bacterial pathogens

TH17 immune reaction is the host tolerable immune reaction against extracellular microorganisms, including extracellular bacteria, fungi, and protozoa. The antigen-presenting cells of TH17 immune reaction are type 2 convention dendritic cells (cDC2), and its innate lymphoid cells are type 3 natural cytotoxic receptor-innate lymphoid cells (NCR-ILC3). The component cells for TH17 immune reaction are neutrophils (N2), IL-17 expressing CD4 T cells, iNKT17 cells, and IgA2 B lymphocytes. The antigen-presenting cells are type 1 myeloid dendritic cells, while the innate lymphoid cells are type3 innate lymphoid cells (NCR-ILCs3). The triggering cytokines are IL-6 and TGF-β, and the master transcription factors are STAT3α, and STAT5α/β. TH17 immune response is associated with type 3 immune complex-mediated autoimmunity.

THαβ immunity is the host immune reaction against infectious particles, including viruses and prions [17,18]. Plasmacytoid dendritic cells are the antigen-presenting cells for THαβ immunity, while IL-10 producing innate lymphoid cells (ILC10) are the innate lymphoid cells. The major immune cells are NK cells (NK1), IL-10–producing CD4 T cells, CD8 T cells (Tc2), and IgG1 B cells, and the initiation cytokines are interferons alpha/beta and IL-10. IL-10 is the central effector cytokine in THαβ immune function, and the master transcription factors are STAT1α, STAT1β, and STAT3β. NK cell with IgG1-mediated antibody dependent cellular cytotoxicity is the effector phase of this immune reaction, causing apoptosis of virus- or prion-infected cell. During apoptosis, DNA fragmentation eliminates viral DNA or RNA, and caspase protein digestion destroys prion pathogenic proteins. Type 2 antibody-dependent cellular cytotoxic autoimmunity is associated with the THαβ immunological pathway.

**Figure 1.**
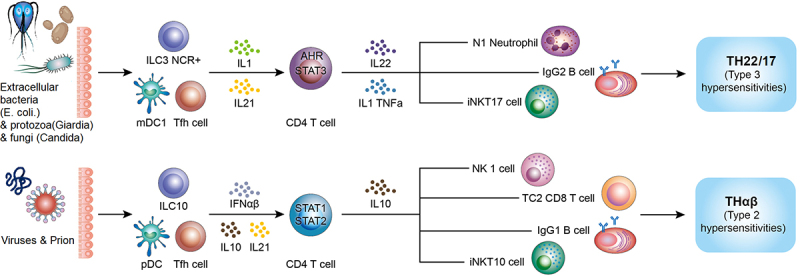
ARDS-related TH17 immunity and anti-viral THαβ immunity. Reprinted from T-H Wen at al. The framework for human host immune responses to four types of parasitic infections and relevant key JAK/STAT signaling IJMS 2021,22:13310.

It is worth noting that host protective immunity against extracellular bacteria is TH17 immune response and host protective immunity against viruses is THαβ immune response. Based on my previous study, pathogens can drive wrong host immunological pathways to induce immune evasion. For example, malarial protozoa can drive TH17 and THαβ immune reactions rather than TH1 immune reaction to cause immune evasion to prevent malarial pathogens from the attack of host immunity. TH1 immune reaction is the host immunological pathway against intracellular protozoa and other intracellular micro-organisms. If there is successful initiation of TH1 immunity, the malarial protozoa can be eliminated immediately after malarial infection. SARS-CoV2 infection has also similar strategy by inducing anti-bacterial TH17 immunity and suppressing anti-viral THαβ immunity to cause immune evasion to let the viral pathogens survive in the host. The figure 1 shows the ARDS related anti-bacterial TH17 immunity and anti-viral immunity.

ARDS is related to anti-extracellular bacterial TH17/TH22 immunity, and virus infection trigger protective THαβ immunity.

## Immune pathogenesis of ARDS: Role of TH17 immunity

ARDS is a detrimental respiratory disorder. Septicaemia is the most significant risk factor for developing ARDS. Sepsis is defined as symptomatic bacteraemia due to bacterial infection. Moreover, PMN overactivity is key in the pathophysiology of ARDS. Thus, extracellular microorganism-induced TH17 immune reaction with PMN overactivity should be a master role in the pathogenesis of ARDS.

The duration sequence of ARDS can be categorized into three stages [19]. First, it is the exudative phase. In this stage, damaged alveolar capillary endothelium and type I pneumocytes induce the loss of the alveolar barrier function and accumulation of protein-rich oedema fluid in interstitial alveolar spaces. Pro-inflammatory cytokines including IL-1, IL-6, and TNF-α and chemokines including IL-8, and leukotriene B4 have been detected in the lung in this stage [20,21]. Neutrophils accumulate in the pulmonary tissues [22,23]. Alveolar oedema can contribute to atelectasis, and hyaline-like change begin to form, followed by intrapulmonary shunting and hypoxia. The condition becomes aggravated by microvascular occlusion, leading to increased alveolar dead space. It also leads to pulmonary hypertension. The exudative stage includes the first week of illness after exposure to predominating ARDS risks containing septicaemia, aspiration pneumonia, lung contusion, near-drowning, toxin, or drug inhalation, major trauma, severe burns, multiple transfusions, acute pancreatitis, and post-cardiopulmonary bypass.

The second stage is the proliferative stage. The duration is from days 7 to 21. Although the majority of patients recover after this phase, some patients suffer from progressive lung injury and early lung fibrosis. In histological findings, this stage involves the initiating of lung repairment, organization of pulmonary exudates, and becoming a lymphocyte-dominant pulmonary infiltration. There is also a multiplication of type II pneumocytes, which can produce lung surfactants and trans-differentiate into type I pneumocytes. Besides, the production of type III collagen precursor, a pulmonary fibrosis marker, is initiated.

The third is the fibrotic stage. Although majority of ARDS patients recover lung function 21 days following the initiation of lung injury, several patients suffer from a fibrotic stage that may require mechanical ventilators for maintaining respiratory function. In histological findings, pulmonary oedema and lung exudates in early stages progress to general interstitial fibrosis. Extensive pulmonary fibrosis can contribute to pulmonary hypertension.

Now, we provide a detailed pathophysiology to explicit the three phases of ARDS. In the first exudative phase, PMNs are recruited to the lung owing to chemotactic molecules, such as IL-8, C5, and leukotriene B4. An infection with bacterium in lung tissue can trigger the activation of lung epithelial cells, lung endothelial cells, lung fibroblasts, and alveolar macrophages during sepsis. Toll-like receptors 1, 2, 4, and 5 and heat-shock proteins (HSP60, HSP70) are essential agents that drive TH17 host immunity [24–32]. Heat-shock proteins are essential reactive proteins in situations described above [33–35]. In particular, HSP60 and HSP70 can activate TH17-related Toll-like receptors. Thus, TH17-associated cytokines, such as IL-17, IL-1, TNF-α, and IL-6, as well as TH17-associated chemokines, such as IL-8 are triggered. Bacterial infection can induce pulmonary epithelial cells to release chemokines and cytokines [36–38]. TH17 cytokines activate TH17 immunity, including the PMN effector function fighting against extracellular bacteria. Thus, the ARDS-related cytokine storm is now understood.

However, the CD86 co-stimulation signal, TCR genes, and most MHC genes are downregulated in ARDS. Thus, adaptive T cells, B cell immunity with specific antibody, and TCR responses against bacteria may not be triggered. However, a previous study detected IL-8 autoantibodies in ARDS patients [39]. IL-8 and leukotriene B4 are the main chemo-attractants in pulmonary tissues [40–46]. This chemokine was originally identified in pulmonary giant cell lines [47]. IL-8 has a high affinity for binding to heparin sulphate and chondroitin sulphate, which are abundant in the lung [48,49]. IL-8 in lung tissues can then recruit PMNs to pulmonary. This can provide reasons why IL-8 produced from distant sites, such as the pancreas, during inflammation can lead to ARDS.

Several defects in the IL8 autoantibody were reported. First, the anti-IL-8–IL-8 complex was detected in the sera of 55% of the healthy controls. There was no significant difference in the number of IL-8–anti-IL-8 complex between ARDS patients and the healthy controls. Second, the IL8 autoantibody can suppress IL-8’s binding activity to neutrophils and chemotaxis activity. Thus, the importance of the IL8 autoantibody in ARDS pathogenesis is doubtful. Moreover, several studies support the role ofTH17 immunity in the pathogenesis of ARDS [21,50]. G-CSF, the growth factor of neutrophils, can cause the common symptoms of ARDS [51]. Moreover, suppression of NFkB can attenuate ARDS progression [52,53]. The key THαβ cytokine, IL-10, also reduces the severity of ARDS [54].

Sepsis is the number one important risk factor for ARDS. However, specific pathogens other than bacterium are also risks for ARDS development. For example, malarial infection can cause ARDS complications. *P. falciparum* can activate HSP60 and HSP70 to trigger TH17 immunity, leading to acute lung injury [55]. SARS-CoV2 and the H1N1 influenza virus can also downregulate anti-viral type 1 interferons and upregulate TH17-like immunity to trigger acute lung injury [7,56]. Thus, TH17 inflammation may be a key pathway in ARDS pathogenesis. It is also noted in burns, major trauma, and acute pancreatic inflammation when TH17-like innate immunity is activated.

In the second proliferative phase, lymphocytes, including TH17-like lymphocytes and Treg lymphocytes, replace PMNs and be the major immune cells in ARDS. TH17-related cytokines, such as IL-17, IL-1, IL-6, and TNF-α, contribute to the inflammation owing to innate immunity activation. However, if bacteria during sepsis or other aetiology suppress the expression of HLA molecules, successful adaptive immune responses with T cell and B cell activation cannot be achieved. One study showed that STAT3, a key transcription factor in TH22/TH17 adaptive immunity, is downregulated. Thus, only the innate immune response is activated in ARDS patients. In addition, activated Treg can secrete TGF-β, which plays a vital role in the pathophysiology of ARDS. Treg cells, the regulatory components of TH17 immunity, are associated with STAT5B activation. So, in the third fibrotic phase, TGF-β-secreting CD4CD25 T helper cells are the dominant immune cells in ARDS [50]. ARDS was induced by bacterial sepsis; thus, failure to induce specific adaptive immunity, such as through TCR and antibodies, cannot successfully overcome bacterial infection. In the early disease stage, overactive innate immunity with PMN activation causes severe lung consolidation, whereas in the later stage, failure of adaptive immunity against bacteria causes abundant regulatory T cells to secrete TGF-β, a powerful fibrosis-promoting agent and the most important and potent stimulant of tissue fibrosis [57,58]. TGF-β promotes the bio-synthesis of collagens [59]; thus, its over-activity in pulmonary tissues causes lung fibrotic change. TGF-β-induced fibrotic change is a process for repairment of cavities caused by bacterial loci, such as abscesses.

The abovementioned pathophysiology can explain the controversial results of many studies. A study found that TLR4 and HSPs can let ARDS become worse [26]. However, other studies reported that TLR4 or HSPs can protect against lung fibrosis after acute lung injury [60–62]. TLR and HSP signals can retain the overactivity of innate inflammatory molecules, such as IL-1. Thus, TGF-β over-activity to promote lung fibrosis is avoided. An animal study reported that neutrophil inhibitors can attenuate the progression of acute lung injury [63]. Thus, TH17 inflammatory processes can fully explain the pathogenesis of ARDS.

Finally, we will mention the two-hit mechanism for Transfusion Related Acute Lung Injury (TRALI). TRALI and ARDS share a common pathogenesis. According to the two-hit hypothesis, TRALI requires the activation of innate immunity, such as CRP, and the production of anti-MHC antibodies. Anti-MHC antibodies may block MHC signalling to prevent the activation of successful adaptive immunity. In addition, downregulated MHC signalling can help trigger the Treg cell components of the TH17 immunological pathway. Thus, overactivated TH17 immunity causes the pathophysiology of ARDS.

### Virus induced ARDS

Although ARDS is usually induced by bacterial infection such as sepsis, ARDS can also be induced by virus infection. For example, influenza virus infection can trigger ARDS as its detrimental complication. Influenza virus-induced ARDS has two important components. First, the viral pathogen can infect respiratory epithelial cells. Second, over-activity of innate immune reaction including TH17 immunity is triggered. Influenza virus infection can induce ARDS by fulfilling the above two conditions. H5N1 and H1N1 are the most identified influenza viruses which can induce ARDS. In a previous study, H5N1 is a potent inducer of proinflammatory cytokines including TNF-α in human macrophages. Thus, TH17 over-activity is associated with H5N1 influenza virus infection. Another paper pointed out that the influenza virus targets lung epithelial cells, damaging tight junctions, and killing infected alveolar cells. Infected epitheliums produce pro-inflammatory cytokines that attract neutrophils as well as macrophages and activate adjacent endothelial cells. Activated endothelial cells and infiltrated leucocytes stimulate further immune cell infiltration, and leucocytes induce production of free radicals like reactive oxygen species to damage lung structure. Activated macrophages also cause trigger apoptosis of epithelial cells. Other virus-induced ARDS has also other components. Adenovirus-induced ARDS is similar to bacteria induced ARDS. However, CD3+ CD4+ T cells are reduced by adenovirus-induced ARDS. It suggests that lymphopenia is also an important finding in virus-induced ARDS. As for Herpesviridae, herpes simplex virus (HSV) and cytomegalovirus (CMV) are the two viral pathogens causing nosocomial viral pneumonia that can progress into ARDS. TH17 related innate immunity over-activation is also related to Herpesviridae viruses caused ARDS [64–66].

Coronavirus can also cause virus-induced ARDS including SARS, MERS, and COVID-19, which is caused by SARS-CoV1, MERS-CoV, and SARS-CoV2, respectively. First of all, these coronaviruses can infect lung epithelial cells to cause alveolar damages. In coronavirus infection, defective type 1 interferon response is noted in SARS, MERS, and COVID-19. Suppression of the interferon alpha/beta is commonly noted in coronavirus infection. SARS-CoV infection in vitro failed to activate interferon regulatory factor 3 (IRF3) which is responsible for producing type 1 interferon. In our previous study, failure of interferon alpha production by SARS-CoV1 is also noted in monocytic cell lines. It is known that eight proteins in SARS-CoV antagonize interferon-stimulated gene (ISG) responses, and these proteins have been identified in MERS-CoV with similar functions. NSP14 and NSP10–16 complex can cap viral mRNAs to protect the SARS-CoV mRNAs from being bound by MDA5 and IFIT1 to trigger IRF activity. The SARS-CoV nucleocapsid protein has an inhibitory effect on type 1 interferon induction, too. The SARS-CoV membrane protein represses type I interferon production by stopping the formation of TRAF3/TBK1/IKK complex. The MERS-CoV membrane protein inhibits the nuclear translocation of IRF3 to stop to activate type I interferon promoter. MERS-CoV ORF4a, ORF4b, ORF5 proteins can all inhibit the nuclear trafficking of IRF3 and activation of the interferon promoter. Type 1 interferons are the initiator of anti-viral THαβ immune response. Thus, coronavirus infection cannot successfully trigger the host anti-viral immunological pathway. The second mechanism of coronavirus-induced ARDS is lymphocyte functional impairment. Recent evidence has found that NK cell number reduction, especially perforin+ NK cell reduction, is noted in COVID-19. The absence of cytotoxic granules containing NK cells also means they are functional impairment to lyse virus-infected host cells. Besides, CD8 and CD4 T cell numbers are also reduced in SARS, MERS, or COVID-19 infection. Lymphopenia is a common finding in coronavirus-induced ARDS. Cytotoxic function of CD8 T cells is also impaired in SARS-CoV2 infection. Thus, adaptive immunity is impaired in coronavirus-induced ARDS. The third mechanism of coronavirus-induced ARDS is over-activation of neutrophils and macrophages. Over-activities of neutrophils and macrophages produce dys-regulated pro-inflammatory cytokines in SARS, MERS, or COVID-19 to induce cytokine storm and ARDS. The over-activated neutrophils and macrophages with massive pro-inflammatory cytokines also link to up-regulated TH17 immune reaction. These mechanisms are the pathophysiology of coronavirus-induced ARDS [67,68].

### Drugs which may be useful to control COVID-19

Due to the above discussion, we know that over-activation of TH17 immunity is related to the pathogenesis of COVID-19. Thus, therapeutic agents targeting TH17 immune reaction may be useful to manage COVID-19 infection. COVID-19 infection is related to up-regulation of anti-bacterial TH17 immunity and down-regulation of anti-viral THαβ immunity. First, we will talk about drugs, which can suppress TH17 immune reaction including silibinin, baricitinib, ruxolitinib, tocilizumab, risankizumab, brodalumab, secukinumab, and netakimab. STAT3 is the key transcription factor to trigger TH17 immunity. Thus, STAT3 inhibitors such as silibinin was used in a pilot study to see its effects on COVID-19 infection in several cancer patients. In this pilot study, pro-inflammatory biomarkers did reduce after silibinin treatment, so STAT3 inhibitor could be useful to control COVID-19. However, the sample size is small, and we cannot make a conclusion. Besides, JAK molecules also play vital roles in triggering STAT3 transcription factor activation during TH17 immunity. The cooperation of JAK1, JAK2, and TYK2 are responsible of interleukin-6 signal transduction to activate STAT3. Preliminary data also show that Baricitinib, a JAK1 and JAK2 inhibitor, is helpful to control COVID-19 infection. Although it was not statistically significant, Baricitinib can reduce COVID-19 mortality in meta-analysis of randomized clinical trials. Ruxolitinib, a JAK1/JAK2/TYK2 inhibitor, has also been suggested to treat COVID-19 infection. However, the efficacy of ruxolitinib was not confirmed in large clinical trials. Combining IL-6 inhibitor tocilizumab and ruxolitinib has been proposed. The non-helpful results of using this strategy could be due to the overlapping mechanism of blocking interleukin-6 signalling of both inhibitors. Thus, no additional effectiveness was observed. On the other hand, it could cause higher adverse effect by overlapping triggering of interleukin-6 signal transduction pathway. It is worth noting that type 1 interferon and interleukin-10 signal transduction relies on JAK1 and TYK2 to trigger THαβ anti-virus host immune reaction. Thus, blocking JAK1 and TYK2 via ruxolitinib can adversely suppress anti-viral host immunity. This can explain why ruxolitinib is not helpful in controlling COVID-19 infection. This can also explain why JAK2 inhibition plays a key role in control COVID-19 infection. As for the other important transcription factor in TH17 immunity, RAR-related orphan receptor inhibitors, the information is limited in COVID-19 patients. Another TH17-related cytokine, interleukin-23, is an interesting drug target. A case report showing that the IL-23 inhibitor risankizumab used in one psoriasis patient can help to control COVID-19 infection. But, information is limited. Interleukin-17 is the central cytokine in the TH17 immunological pathway. Brodalumab is an IL-17 receptor inhibitor, and secukinumab is an IL-17 inhibitor. Both drugs are suggested to be used to control COVID-19 infection. Nevertheless, we still need randomized clinical trials to confirm the efficacy of the two drugs. The other IL-17 inhibitor netakimab has been tested in a pilot clinical trial. After 3 days of therapy, body temperature, SpO2/FiO2, and CRP improved significantly. However, the ICU transfer rate, need for mechanical ventilation, and one-month mortality rate was statistically non-significantly reduced. A statistically significant decrease in the duration of hospitalization in the netakimab group was observed. These above evidences suggest TH17 immunity inhibition can be helpful in the management in COVID-19 infection. However, the clinical guideline already includes IL-6 inhibitor, a key TH17 initiator, to treat COVID-19 with complications. We will need new drug candidate not only to control TH17 related pro-inflammatory cytokine storm but also to successfully initiate anti-viral THαβ immunity to suppress viral infection. Interleukin-10 can play a vital role [69–72].

### IL-10 as host immunity factor against viruses

The host immunological pathway against viruses is the THαβ immunity [18]. The THαβ immune response is driven by type 1 interferon and IL-10. Pegylated IL-10 (pegilodecakin) is the drug form of IL-10 [73]. It has been used in the cancer clinical trial owing to its immune stimulatory effects. The main T helper cells of THαβ immunity are IL-10-CD4 T cells, formerly called Tr1 cells [74]. The major effector cells of THαβ immunity are IL-10-CD4 T cells, NK cells, CD8 T cells, and IgG1-producing B cells. CD8 T cells and NK cells have an antibody-dependent cellular cytotoxicity (ADCC) function, which induces the apoptosis of virus-infected cells, leading to viral and cellular DNA fragmentation and elimination of viruses. Type 1 interferon is the main cytokine that triggers anti-viral immune responses. Because SARS-CoV can downregulate type 1 interferon production to confer the virus survival advantage, type 1 interferons (e.g. interferon-α and interferon-β) have been used to treat SARS infections [75,76].

IL-10 is the key cytokine in the THαβ immune response and a potent anti-viral cytokine [74]. Previous studies have showed that IL-10 can suppress viral infections, including the diseases caused by HSV, alpha virus, HIV, RSV, HCV, Japanese encephalitis virus, influenza virus, vaccinia virus, dengue virus, and coronavirus [77–87]. IL-10 can also reduce coronavirus-induced CNS demyelination or encephalomyelitis [88–92]. Several pox viruses, such as the EBV or CMV, encode viral IL-10 (vIL-10) to mimic cellular IL-10 or serve as a decoy [93,94]. Thus, virus can escape the host immune response through vIL10 [95–98]. These viral encoded IL-10 lack the functions of stimulating thymocyte or PBMC proliferation and B cells class II MHC up-regulation which are normal functions of cellular IL-10 [93–95,97]. Viral encoded IL-10 also suppresses the induction of type 1 interferons from plasmacytoid dendritic cells and prevents the expression of co-stimulating molecules on lymphocytes [99,100]. Type 1 interferons (IFNα & IFNβ) can drive naïve CD4 T cells to become IL-10 producing CD4 T cells, formally called Tr1 cells. IL-10 producing CD4 T cells are the major T helper cells in antiviral THαβ immunity. IL-10 was originally clones as a T cell growth factor. IL-10 can activate the activities of NK cells, B cells, and CD8 T cells [101–104]. IL-10 producing CD8 T cells are the main component cells in anti-viral THαβ immunity. It can also cause B cell antibody isotype switch to IgG1 antibody, which is the anti-viral antibody [18,105]. Lymphocytes including NK cells, CD8 T cells, and IgG1 B cells are main effector cells in anti-viral host immunity. IL-10 is not a pure immunosuppressant because of these functions. IL-10 is also an immunostimulant to trigger host immune reaction against viruses. These findings suggest that IL-10 plays an important role in anti-viral immunity.

TH3 cells, which are detected in chronic virus infection, are characterized by co-secretion of IL-10 and TGF-β [105]. Blackburn and Brooks suggested IL-10 is related to persistent viral infection; however, they neglected the role of TGF-β and IL-10 co-expressing TH3 cells [106,107]. A previous study pointed out that TGF-β signalling contributes to the persistence of lymphocytic choriomenigitis virus (LCMV) infection in mice [108]. In fact, IL-10 exerts a potent anti-viral activity by activating NK cells and CD8 T cells [101]. TGF-β can weaken the anti-viral effect of IL-10 and mildly control chronic virus infections to avoid fulminant viral diseases, such as viral hepatitis [105]. In fact, based on the findings of Blackburn and Brooks, we speculate that the actual cause of persistent virus infection is TGF-β and not IL-10. A figure of showing anti-viral TH

### IL-10 as an immune-modulation mediator

IL-10 possesses a strong immune-modulation activity [109] and is considered as an anti-inflammatory cytokine that can suppress pro-inflammatory cytokine production by macrophages [110–113]. IL-10 can also suppress the functions of neutrophils [114,115]. Previous studies have shown IL-10 can suppress the pathogenic TH17-like innate immunity to ameliorate autoimmunity [116–120]. THαβ CD4 T cells can secrete IL-10, and IL-10 can activate CD8 T cells and NK cells, causing immunity against virus infection. Thus, IL-10 has a dual role in anti-viral immunity: suppression of macrophages and neutrophils and activation of CD8 T cells and NK cells for anti-viral immunity [101].

One study reported elevated IL-10 levels in patients with COVID-19 with ARDS; however, whether this serves a protective or harmful role remains unknown [4]. ARDS is related to overactivation of macrophages and neutrophils and manifests as acute lung injury caused by hyperactivity of innate immunity. Indeed, previous studies have showed that IL-10 from Tr1 cells (now called THαβ cells) can ameliorate ARDS in some animal models [54,121]. In addition, SARS-CoV inhibited by CD4 T cells and CD8 T cells in animal models were reported [90,91]. Thus, CD4 T cells secrete IL-10, which subsequently activate CD8 T cells [101]. Overall, IL-10 may suppress coronavirus infection.

TRALI is considered as the same syndrome as ARDS. They have common clinical manifestations. A two-hit hypothesis was proposed for the aetiology of TRALI. First, over-activity of innate immunity is triggered happens. Second, there is the presence of anti-HLA antibodies. In TRALI, the level of IL-10 is usually low, with bad prognosis [122]. A previous study found that TRALI can be treated using IL-10 [123]. Several studies have reported that IL-10 can treat acute lung injury or pulmonary inflammation due to viruses, including influenza virus or respiratory syncytial virus (RSV) [64,82]. Thus, ARDS may also be treated by IL-10 [123]. IL-10 plays a strong protective role in a viral infection of the lung [79]; thus, it may be a good candidate for virus-induced ARDS treatment.

### IL-10 as an anti-fibrotic agent

IL-10 has a potent anti-fibrotic activity. It may compete with other pro-fibrotic cytokines, such as TGF-β, to inhibit fibrosis. Several previous studies have found that IL-10 can suppress the activity of fibroblasts [124,125] and ameliorate pulmonary fibrosis [126–129]. The common sequalae of ARDS is pulmonary fibrosis. Overall, we can infer that the use of IL-10 can treat SARS-CoV-2–induced ARDS and pulmonary fibrosis through the anti-viral THαβ immunological pathway, which was previously discussed [105].

### Possible overdose and resistance of IL-10 therapy

Previous studies showed that IL-10 injection in healthy voluneters is safe and well tolerated. The administated dosages are 1.0, 2.5, 5.0, 10, 25, 50 ug/kg [130–133]. At high dosage of interleukin-10 administration, adverse effects include flu-like syndrome, mild to moderate decrease of neutrophil counts, mild decrease of lymphocyte count, and mild delayed decrease of platelet counts. Pegylated interleukin-10 can protect itself from metabolizing and prolong the half-life of the drug [134]. Overall, interleukin-10 administration is safe and well tolerated. As for the resistance of interleukin-10, observation found that high glucose in blood circulation is associated with interleukin-10 resistance [135]. However, the detailed mechanism is unclear. In addition, some viruses, such as EBV or CMV encode the viral interleukin-10. Viral interleukin-10 can compete the binding to host interleukin-10 receptor, so it might lead to host interleukin-10 resistance [93–97]. Viral interleukin-10 can still have immuno-suppression effects of interleukin-10, but it abrogates the anti-viral and immuno-stimulating effects of interleukin-10. That is the reason why these viruses can have the phenomenon of immune evasion.

### Responses to previous studies

Several previous studies have pointed out that IL-10 levels are elevated after SARS-CoV2 infection. This elevation may be related to the severity of COVID-19. In addition, the levels of pro-inflammatory cytokines, especially IL-6, were more highly elevated in severe cases of COVID-19 [136]. Thus, those authors concluded that IL-10 may be related to worse prognosis of SARS-CoV2 infection [137–139]. Furthermore, those authors suggested that IL-10 may play a role in the pathogenesis of COVID-19. In fact, there is another two papers, which pointed out down-regulation of IL-10 during COVID-19 infection and higher level of IL-10 was seen in recovered patients compared to severe COVID-19 patients [140,141]. Another two studies also had findings against IL-10 elevation in COVID-19 [142,143]. Thus, observations about elevation of IL-10 serum levels during COVID-19 infection are not consistent. A previous study found that IL-10 can be effective to treat solid tumours, suggesting that it is a pro-inflammatory cytokine [73]. However, this conclusion is questionable. IL-10 potently suppresses cytokine storms with elevations of pro-inflammatory cytokines. IL-10 is effective against solid tumours because it is an anti-viral cytokine. Thus, IL-10 has a similar mechanism in combating solid tumours as oncolytic viruses.

Nevertheless, previous studies have some limitations and false interpretations. IL-10 levels can be reactively elevated during COVID-19. Interleukin-10 levels may not be elevated enough to combat severe disease during COVID-19. A study showed that the IL-6/IL-10 ratio is a more ideal prognosis biomarker for COVID-19. Other studies have also suggested that this ratio could be a prognostic indicator for systemic inflammatory diseases [144–146]. Thus, it should be noted that higher levels of IL-6 and lower levels of IL-10 are related to COVID-19 severity and worse prognosis. IL-10 is a key cytokine during THαβ anti-viral immunity, and its production is triggered by type 1 interferons. However, SARS-CoV2 can downregulate type 1 interferons and prevent the induction of IL-10. In fact, IL-10 plays a beneficial role against coronavirus infection. Current clinical guidelines for treating COVID-19 with ARDS is triple therapy: remdesivir, tocilizumab (anti-IL-6 monoclonal antibody), and corticosteroid [147]. Remdesivir is the anti-SARS-CoV2 agent. Tocilizumab is the monoclonal antibody to suppress pro-inflammatory cytokine: IL-6. Corticosteroid is the potent immunosuppressant to suppress ARDS with cytokine storm in COVID-19. Corticosteroid can totally suppress host immune responses, including adaptive immune suppression with lymphocyte inhibition. Thus, it can cause host antiviral immunity to become impaired. Thus, it is not safe to use corticosteroid to treat SARS-CoV2-induced ARDS in COVID-19. IL-10 can inhibit all pro-inflammatory cytokines, including IL-1, IL-6, IL-8, and TNF-α and it can stimulate anti-viral immune reaction with activating CD8 T cells, NK cells, and IgG1 B cells activities. Thus, it is more logical to use IL-10 to treat COVID-19 instead of corticosteroid.

### Other THαβ cytokines to control of COVID-19 infection

There are other THαβ cytokines that can also be important to control COVID-19 infection. SARS-CoV can downregulate type 1 interferons. Thus, type 1 interferons could be used to control COVID-19. Additionally, type 3 interferons have a similar anti-viral mechanism as type 1 interferons [148], and SARS-CoV2 can be suppressed by type 3 interferons [149]. Thus, type 3 interferons can also be used to control SARS-CoV2. In addition, IL-27 is a cytokine that upregulates IL-10 [150]. Thus, the anti-viral ability of IL-27 makes it a potential therapeutic agent for COVID-19 [151,152]. These cytokines may serve as possible management strategies to treat COVID-19.

## Conclusions

To date, there is no effective therapeutic agent for COVID-19 with ARDS. The success rate of IL-6 inhibitors for COVID-19 treatment is only 32%, which does not satisfy the clinical needs. This review proposes IL-10 as a new therapeutic strategy for COVID-19 with ARDS. We discussed its treatment potential based on the three mechanisms. First, it is a key anti-viral infection cytokine that activates CD8 T cells and NK cells. Second, it exhibits immune modulation activity by suppressing macrophages and neutrophils to produce pro-inflammatory cytokines. Third, IL-10 is a potent anti-fibrotic agent that competes with pro-fibrotic cytokines, such as TGF-β, to inhibit pulmonary fibrosis. Thus, we suggest that IL-10 is a potential therapeutic candidate for COVID-19 with ARDS, especially in SARS-CoV-2 infection complications with ARDS.

## Data Availability

Data sharing not applicable-no new data generated
